# Clinical and radiological outcome of conservative vs. surgical treatment of atraumatic degenerative rotator cuff rupture: design of a randomized controlled trial

**DOI:** 10.1186/1471-2474-12-25

**Published:** 2011-01-26

**Authors:** Frederik O Lambers Heerspink, Roy AG Hoogeslag, Ron L Diercks, Pepijn JM van Eerden, Inge van den Akker-Scheek, Jos JAM van Raay

**Affiliations:** 1 Martini Hospital Groningen Department of Orthopaedic Surgery PO 30 0033 9700RM Groningen, The Netherlands; 2 Zorggroep Twente Department of Orthopaedic Surgery PO 7600 7600 SZ Almelo, The Netherlands; 3 University Medical Center Groningen Department of Orthopaedic Surgery PO 30 001 9700 RB Groningen, The Netherlands; 4 University Medical Center Groningen Department of Radiology PO 30 001 9700 RB Groningen, The Netherlands

## Abstract

**Background:**

Subacromial impingement syndrome is a frequently observed disorder in orthopedic practice. Lasting symptoms and impairment may occur when a subsequent atraumatic rotator cuff rupture is also present. However, degenerative ruptures of the rotator cuff can also be observed in asymptomatic elderly individuals. Treatment of these symptomatic degenerative ruptures may be conservative or surgical. Acceptable results are reported for both treatment modalities. No evidence-based level-1 studies have been conducted so far to compare these treatment modalities. The objective of this study is to determine whether there is a difference in outcome between surgical reconstruction and conservative treatment of a degenerative atraumatic rotator cuff tendon rupture.

**Methods/Design:**

A randomized controlled trial will be conducted. Patients aged between 45 and 75 with a symptomatic atraumatic rotator cuff rupture as diagnosed by MRI will be included. Exclusion criteria are traumatic rotator cuff rupture, frozen shoulder and diabetes mellitus. Patients will be randomized into two groups. Conservative treatment includes physical therapy according to a standardized protocol, NSAIDs and, if indicated, subacromial infiltration with a local anesthetic and corticosteroids. Surgical reconstruction is performed under general anesthesia in combination with an interscalenus plexus block. An acromioplasty with reconstruction of the rotator cuff tendon is performed, as described by Rockwood et al. Measurements take place preoperatively and 6 weeks, 3 months, 6 months and 1 year postoperatively. The primary outcome measure is the Constant score. Secondary measures include both disease-specific and generic outcome measures, and an economic evaluation. Additionally, one year after inclusion a second MRI will be taken of all patients in order to determine whether extent and localization of the rupture as well as the amount of fatty degeneration are prognostic factors.

**Discussion:**

Both surgical as conservative treatment of a symptomatic atraumatic rotator cuff tendon rupture is used in current practice. There is a lack of level-1 studies comparing surgical vs. conservative treatment. This randomized controlled trial has been designed to determine whether the surgical treatment of a degenerative atraumatic rotator cuff tendon rupture may lead to a better functional and radiological outcome than conservative treatment after one year of follow-up.

**Trial registration number:**

Netherlands Trial Register (NTR): NTRTC2343

## Background

Subacromial impingement syndrome (SIS) is the most frequently recorded disorder of shoulder complaints [1-3]. The etiology of SIS is multifactorial. Both extrinsic factors, like impingement of the rotator cuff relative to the acromion, age, smoking and diabetes mellitus, and intrinsic factors, like overhead use, repetitive microtraumata and inflammation pathways, contribute to the development of SIS [4]. This leads to a continuum of subacromial edema and subdeltoideal bursitis, and eventually an atraumatic rupture of the rotator cuff [5].

Clinically, a rotator cuff rupture is characterized by painful or impaired active abduction, with reduced strength in abduction, external rotation and elevation. However, a degenerative rotator cuff rupture may also be asymptomatic [6]. Ten percent of the population has an atraumatic and subclinical rotator cuff rupture in their fourth decade of life; this increases to 50% in the sixth decade and 80% in the eight decade [7]. Fifty percent of patients over 50 years of age with an asymptomatic rotator cuff rupture become symptomatic within 5 years [8].

Treatment of symptomatic degenerative rotator cuff ruptures can be conservative or surgical [9,10]. Objectives are relieving pain and restoring shoulder function. Conservative treatment consists of analgesic medication, such as non-steroidal anti-inflammatory drugs (NSAIDs), subacromial infiltration of lidocaine and corticosteroids, and physical therapy. The few existing studies that involve conservative treatment have not properly defined what this non-surgical treatment entailed [11-15]. Described success rates of conservative treatment vary widely, from 40 to 80% [11-15]. The quality of the studies that support conservative management is generally poor. Most studies are of a low-quality level, are retrospective, and do not represent the entire population of patients with a rotator cuff rupture. Moreover, they do not distinguish between degenerative atraumatic and traumatic rotator cuff ruptures [11-15].

Surgical treatment for rotator cuff ruptures includes subacromial bursectomy, acromioplasty, debridement in case of partial tears, and surgical reconstruction of the rotator cuff. A combination of procedures is often carried out, which can be performed as open procedures or using arthroscopic techniques [16].

Reported satisfactory outcome of surgical treatment of a rotator cuff rupture is 38-95% [17-22]. Most studies do not distinguish between degenerative atraumatic and traumatic rotator cuff ruptures. In some studies the satisfaction rate as reported by patients was high, yet functional outcome was poor [19,20,22]. This seems to indicate that pain is more important than range of motion from a patient perspective. Duration of symptoms longer than 34 months, female gender and higher ASA scores are correlated with a poor outcome [19]. Postoperative retears occur in 11-92% of patients [23]. A rotator cuff retear correlates with decreased functional outcome [24]. It is stated that the amount of fatty muscular degeneration and atrophy of the rotator cuff is a bad prognostic factor for functional outcome [25].

A recent review could not draw firm conclusions with regard to efficacy of reconstruction of rotator cuff ruptures [10]. A significant number of patients underwent surgery after nonoperative therapy, suggesting that they may not have been satisfied with the latter. Currently there are no level-1 randomized studies that compare surgical to non-surgical treatment. It is therefore unclear whether surgical repair of a symptomatic atraumatic full-thickness rotator cuff rupture of the shoulder leads to a better functional result than conservative treatment.

We designed a randomized controlled trial aiming to compare clinical, functional and radiological outcome of surgical reconstruction versus conservative treatment of atraumatic degenerative rotator cuff ruptures. Moreover, it will be determined whether extent and localization of the rupture as well as the amount of fatty degeneration are prognostic factors for the outcome of both treatments. This paper reports the study design of the COPACABANA (**C**onservative vs. **Op**erative treatment of **A**traumatic rotator **C**uff rupture, **A**natomically and **R**adiology **A**fter o**N**e ye**A**r) study.

## Methods/Design

### Design

The COPACABANA study is designed as a level-1 prospective randomized controlled trial. The study design, procedures, protocols and informed consent are approved by the local Medical Ethical Committee (registration number MZH2008-36). The trial is registered in the Netherlands Trial Registry (NTR TC 2343). The inclusion period is planned from April 2009 to December 2011.

### Study population

An MRI scan will be done on all patients aged between 45 and 75 with a clinically suspected atraumatic rotator cuff rupture who were referred to the departments of orthopedic surgery and rehabilitation of both Martini Hospital and University Medical Center Groningen, The Netherlands. If the MRI of the affected shoulder shows - as assessed by two independent assessors (PE, FOLH) - a full thickness rotator cuff rupture with degenerative characteristics, the patient will be included in the study. Exclusion criteria are traumatic rotator cuff rupture, previous surgical treatment of the shoulder, frozen shoulder, radiological and symptomatic osteoarthritis of the glenohumeral or acromioclavicular joint, (rheumatoid) arthritis, diabetes mellitus and cognitive disorders, neurological disease or language barriers impairing participation. All patients must give informed consent before participating in the COPACABANA study.

### Randomization

Patients who meet the inclusion criteria will be informed about the study. After consenting to participate, patients are allocated to conservative or surgical treatment. One researcher (FOLH) will perform randomization. Randomization procedure is based on opaque sealed envelopes. Patients will be informed in accordance with the Dutch Medical Treatment Contracts Act about the treatment, the risk of complications and the anticipated period of recovery for both treatment groups.

### Interventions

Conservative treatment consists of a subacromial steroid infiltration. The first infiltration will be performed immediately following inclusion. Using a 21-gauge needle with the covered syringe, the treating orthopedic surgeon injects the patient's subacromial bursa via the anterolateral approach applying an aseptic technique[1]. If no relief is obtained with the first subacromial infiltration, a second infiltration will be performed under radiological or ultrasound guidance. The infiltration will be repeated at a maximum of three times. Further treatment consists of analgesic medication with NSAIDs, paracetamol, and/or tramadol. Patients will be referred to a physiotherapist that uses a standardized protocol developed by the Department of Physical Therapy of Martini Hospital (Table 1). During the first 4 weeks passive movements will be performed to preserve glenohumeral and scapulothoracic mobility. After 6 weeks active movements will be performed. Twelve weeks after commencement of treatment physiotherapy will be aimed at strength regeneration. Mobility will be further optimized. Physical therapy is continued until patients reach a full range of motion and an improvement in strength is achieved.

**Table 1:** Protocol for conservative treatment

Weeks 0-4
Maintain scapulothoracic mobility. Passive anteflexion/abduction 45°, exorotation 10°. Circumduction training.

**Weeks 4-6**

Guided active motion.

**Weeks 6-12**

Active motion guided by pain. Active coordination and stability training.

**Weeks 12--**

Start strength training. Optimize mobility, coordination and stability training

The surgical procedure is performed by two qualified and experienced surgeons (JvR, RD). Surgery will take place within 6 weeks after the inclusion date. If indicated, NSAIDs or other pain medications will be prescribed in the period until surgery.

Surgery is carried out under general anesthesia, if indicated supplemented with an interscalenus plexus block. The operation is performed in beach-chair position. The approach applied is the anterolateral "deltoid on" approach. The coracoacromial ligament is detached from its insertion and the subacromial bursa is excised. The anteroinferior part of the acromion is removed, as well as osteophytes on the lower surface of the acromioclavicular joint. The ruptured ends of the rotator cuff are mobilized and repaired. Reconstruction depends on the type of rupture [26], augmented by using bone anchors. If repair is not possible due to extensive degenerative changes, debridement and a biceps tenolysis at the glenoid and tenodesis in the sulcus bicipitalis tenodesis with bone anchors will be performed. No tendon transfers or artificial augmentation devices will be used. The deltoid muscle is reattached to the acromion by transosseal refixation. As no differences in outcome have been reported between open or arthroscopic repairs, we chose to use the open approach, omitting the possible bias of a learning curve[16]. Standard postoperative pain relief protocols used in the participating hospitals will be used throughout the study.

After surgery, the patient receives a sling for six weeks combined with exercise instructions (Table 2). Patients will be referred to a physiotherapist who uses a standardized protocol (Table 2). During the first 6 weeks the emphasis is on mobility, not on strength. After 6 weeks strength development will be included as well. Physical therapy after surgery is done until patients reach a full range of motion and an improvement in strength is achieved.

**Table 2:** Protocol for surgical treatment

**1**^**st **^**day postoperatively**
Active motion of elbow, wrist, hand. Passive shoulder motion up to pain threshold. Affected arm in sling for 6 weeks; remove only for personal hygiene.

**3**^**rd **^**day postoperatively**

Passive motion (including passive exorotation). Submaximal isometric contractions (<30% of voluntary isometric force). Practice ADL* and sleep postures.

**2 weeks postoperatively**

Outpatient control for wound, neurovascular status, etc. Control of motion and exercises. Continuation of exercises. No combined abduction-exorotation.

**6 weeks postoperatively**

Outpatient control. Goal: passive motion is the same as preoperative motion. Start guided active motion and expand to active motion. Remove sling. Start rotator cuff rehabilitation. No combined abduction-exorotation.

**8 weeks postoperatively**

Goal: range of active motion is at least 50% of preoperative.

**12 weeks postoperatively**

Outpatient control. Goal: active range of motion is the same as preoperative. Start combined abduction-exorotation movement.

* activities of daily living

### Measurements

**Figure 1:**
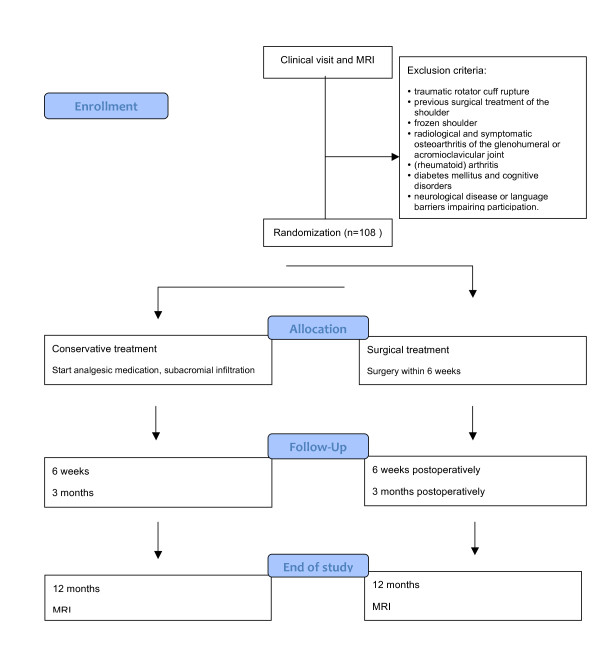
Flow diagram for COPACABANA study

Outcome assessment will take place in both study groups at randomization (T0), 6 weeks post-randomization (T1), and at 3, 6 and 12 months (T2, T3, T4) post-randomization. At 12 months follow-up a second MRI of the affected shoulder will be taken (Figure 1). The outcome assessment will take place in an outpatient clinic setting and will focus on shoulder function and pain. Functional outcome (primary outcome) will be determined with the Constant Murley score. Secondary outcome measures are the Dutch Simple Shoulder Test, the Visual Analogue Scale for pain and restriction, the Goutallier score (Table 3), anatomical localization of the rotator cuff rupture and integrity of the rotator cuff. Further, a generic, patient-based cost-effectiveness analysis will be performed.

**Table 3:** Criteria for grading muscle fatty degeneration (Goutallier score)

Grade 0	No fatty deposits
Grade 1	Some fatty streaks
Grade 2	More muscle than fat
Grade 3	As much muscle as fat
Grade 4	Less muscle than fat

#### Shoulder function and clinical shoulder score

The Constant Murley Score will be used [27]. This score system combines a shoulder function test (65 points) with a subjective evaluation of shoulder complaints by the patient (35 points). Additionally, patients will complete the Dutch Simple Shoulder Test; a questionnaire of 13 questions (yes/no) relating to the perception of symptoms of shoulder pain and function in the last 24 hours [28].

#### Pain

Pain and restriction will be measured using a Visual Analogue Scale [29]. Zero represents the least likely pain and restriction, and 10 the most likely pain and restriction.

#### Radiological investigation

The Goutallier score (Table 3), which classifies the amount of fatty degeneration of a muscle and anatomical localization of the rotator cuff rupture, will be reviewed on an MRI [30,31]. The MRI will be made at inclusion and 12 months after commencement of treatment. The extent and localization of the rupture as well as the amount of fatty degeneration and amount of retraction is determined by two independent assessors (PE, FOLH).

### Sample size

Different prospective studies on clinical outcomes following open rotator cuff repair in full-thickness rotator cuff ruptures state that the Constant Murley Score 1 to 3 years postoperatively is between 70 and 90 [19,31-34]. Sample size calculation was performed; primary outcome measure was the Constant-Murley Score, whereby 10 points was considered a clinically relevant difference between the two groups, with a standard deviation of 20, alpha set on 5% and power on 80%. This resulted in a required number of 49 patients in each group. Assuming a dropout rate of 10%, two groups of 54 patients will have to be included.

### Statistical analysis

The means and standard deviations of the intervention and control group will be calculated for patient and outcome characteristics. In order to determine whether a difference exists between the surgical and conservative groups as far as primary outcome measurement (pain and function of the shoulder as determined with the Constant-Murley Score) over all five measurement moments is concerned, a random effect analysis shall be carried out which includes corrections for hospital and operating surgeon and for a number of patient characteristics. This analysis will be repeated with the secondary outcome measurements as dependent variables.

The prognostic value of localization and extent of the rotator cuff rupture and the degree of fatty degeneration influencing the functional recovery after 12 months will be analyzed using a linear regression model. Here too corrections will be made for hospital and operator and for a number of patient characteristics.

## Discussion

Both conservative and surgical treatment for degenerative atraumatic rotator cuff tendon rupture is performed in current practice. A clear distinction between indications for surgical repair and conservative treatments cannot be made on the basis of the current evidence. To date, there are no randomized controlled studies that compare outcome of surgical vs. conservative treatment of atraumatic rotator cuff ruptures. The COPACABANA study is designed to determine which treatment result in better outcome. Furthermore, it is possible to identify prognostic factors for outcome of surgical repair of the rotator cuff.

## Competing interests

The authors will not receive any reimbursements, fees or salary for conducting the study.

## Authors' contributions

RH, RLD and JVR originated the idea for the study, contributed to its design with IvdA and developed the intervention protocol. FOLH is responsible for the data collection. RH and FOLH drafted the manuscript. All authors read, edited and approved the final manuscript.

## Pre-publication history

The pre-publication history for this paper can be accessed here:

http://www.biomedcentral.com/1471-2474/12/25/prepub
